# Sexual dysfunction and motor disability in Parkinson’s disease: any link?

**DOI:** 10.1192/j.eurpsy.2023.2327

**Published:** 2023-07-19

**Authors:** A. A. Mousli, R. Zouari, A. Lahmer, S. zakaria, A. Rachdi, F. Nabli, S. Ben Sassi

**Affiliations:** 1 Neurology, the National Institute of Neurology Mongi Ben Hmida, ariana, Tunisia

## Abstract

**Introduction:**

Parkinson’s disease (PD) is a chronic, neurodegenerative disorder leading to dopamine deficiency. Phenotypically, there is a wide spectrum of motor and non-motor symptoms (NMS). Among NMS, sexual dysfunction (SD) is one of the most disabling and crippling symptom. However, SD are usually neglected and underdiagnosed in PD patients.

**Objectives:**

Our study aimed to estimate the effect of motor disability and the disease course on sexual dysfunction in PD patients.

**Methods:**

This retrospective study included 42 patients (18 males and 24 females) from the department of neurology of the National Institute of Neurology Mongi Ben Hmida in Tunis, Tunisia, diagnosed with PD between 1999 and 2022. The diagnosis of PD was confirmed according to the Movement Disorder Society (MDS) diagnostic criteria of PD. The MDS Unified Parkinson’s Disease Rating Scale (MDS-UPDRS) motor was used to estimate motor disability and Hoehn and Yahr (H&Y) stage was used to rate disease severity. The SD of PD patients was measured by applying the sexual items of Scales for Outcomes in Parkinson’s Disease - Autonomic Dysfunction (SCOPA-AUT).

**Results:**

SD was observed in only 11 patients (26.2%) with a sex-ratio of M/F = 1.2 and a mean age of 52 (between 40 and 72). The mean age of PD onset was 47. According to the MDS-UPDRS part III, 1 patient had a severe motor disorder (MDS-UPDRS> 59), and according to the H&Y scale, no patient had a severe stage of the disease. Nine patients had motor complications such as motor fluctuations and L-Dopa induced dyskinesia.

The SD described by our patients were: women reported Vaginal Dryness (4 patients), with difficulties reaching an orgasm (3 patients); men reported erectile dysfunction (6 patients), and difficulties in reaching an orgasm (6 patients). Among these patients, 3 were treated for SD with Tadalafil (all males).

In our study, no significant gender-related differences were found in scores related to SD in patients with PD. Neither the disease severity nor the motor disability was significantly associated to sexual disorders (respectively p=0.26 and p=0.12). Also, Motor complications induced by L-Dopa medication, assessed by the part IV of MDS-UPDRS scale, had no significant effect in the occurrence of SD in PD (p=0.78).

**Conclusions:**

Sexual behavior has neuronal and hormonal modulation. Lack of dopamine seems to have an important role in the development of SD. However, it occurs independently of the disease severity and the motor disability. Thus, clinicians should be aware of the importance of assessing and treating such symptoms since the beginning of the disease.

**Disclosure of Interest:**

None Declared

